# Advances in the Mechanisms Affecting Meniscal Avascular Zone Repair and Therapies

**DOI:** 10.3389/fcell.2021.758217

**Published:** 2021-10-28

**Authors:** Wenqiang Yan, Wenli Dai, Jin Cheng, Yifei Fan, Tong Wu, Fengyuan Zhao, Jiahao Zhang, Xiaoqing Hu, Yingfang Ao

**Affiliations:** ^1^Department of Sports Medicine, Peking University Third Hospital, Beijing, China; ^2^Institute of Sports Medicine of Peking University, Beijing, China; ^3^Beijing Key Laboratory of Sports Injuries, Beijing, China

**Keywords:** meniscus, meniscal injuries, avascular zone, biological mechanisms, biological augmentation

## Abstract

Injuries to menisci are the most common disease among knee joint-related morbidities and cover a widespread population ranging from children and the general population to the old and athletes. Repair of the injuries in the meniscal avascular zone remains a significant challenge due to the limited intrinsic healing capacity compared to the peripheral vascularized zone. The current surgical strategies for avascular zone injuries remain insufficient to prevent the development of cartilage degeneration and the ultimate emergence of osteoarthritis (OA). Due to the drawbacks of current surgical methods, the research interest has been transferred toward facilitating meniscal avascular zone repair, where it is expected to maintain meniscal tissue integrity, prevent secondary cartilage degeneration and improve knee joint function, which is consistent with the current prevailing management idea to maintain the integrity of meniscal tissue whenever possible. Biological augmentations have emerged as an alternative to current surgical methods for meniscal avascular zone repair. However, understanding the specific biological mechanisms that affect meniscal avascular zone repair is critical for the development of novel and comprehensive biological augmentations. For this reason, this review firstly summarized the current surgical techniques, including meniscectomies and meniscal substitution. We then discuss the state-of-the-art biological mechanisms, including vascularization, inflammation, extracellular matrix degradation and cellular component that were associated with meniscal avascular zone healing and the advances in therapeutic strategies. Finally, perspectives for the future biological augmentations for meniscal avascular zone injuries will be given.

## Introduction

Menisci, semilunar fibrocartilage tissue located between the femoral condyle and tibial plateau, function in load-bearing, load transmission, shock absorption, lubrication and nutrition during dynamic movements of the knee (Cameron and Macnab, 1972; Newman et al., 1989; Proctor et al., 1989; Zhu et al., 1994; Kohn and Moreno, 1995; Tissakht et al., 1996; Makris et al., 2011). Meniscal injuries are the most common disease within knee joint related injuries and partial meniscectomies are the most frequent surgeries performed by orthopedic surgeons in the United States (Englund et al., 2008). A previous epidemiologic study suggested that the prevalence of meniscus injuries in the right knee ranged from 19% [95% confidence interval (CI), 15–24] among women aged 50–59 years to 56% (95% CI, 46–66) among men aged 70–90 years (Englund et al., 2008). Moreover, meniscal pathology has a higher prevalence in athletes (Yeh et al., 2012; Beals et al., 2016). Hence, the incidence of meniscal injuries covers a widespread population ranging from children and the general population to the old and athletes.

**FIGURE 1:**
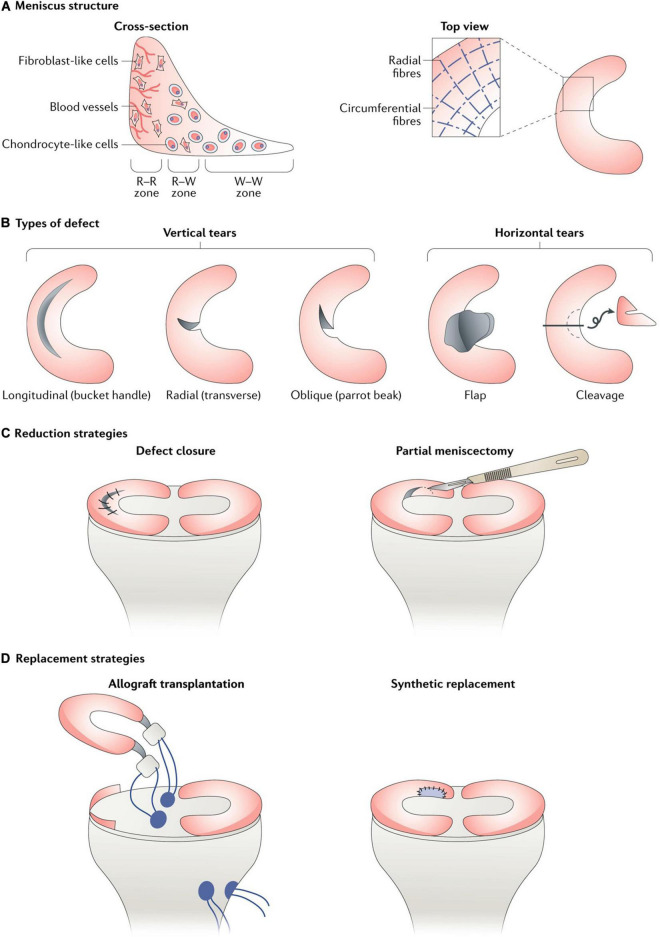
**(A)** Meniscus consists of three main zones: red–red (R–R), red–white (R–W) and white–white (W–W). The R–R zone is fully vascularized and the W–W zone is avascular; **(B)** A variety of different types of defect can occur in the meniscus, some of which are easier to repair than others owing to their intrusion into vascular or avascular zones; **(C)** Reduction strategies in current use include defect closure with sutures or anchors and the trimming of torn pieces (partial or total meniscectomy); **(D)** Replacement strategies in current use include allograft transplantation and the use of synthetic implants. This figure was adapted permission from Kwon et al. (2019).

Vascularization in meniscal tissue varies in different developmental stages. The whole meniscus is richly vascularized from the prenatal period to shortly after birth. Afterward, vascularization concentrates in the peripheral (10–30%) at 10 years of age. The blood supply is present solely in the peripheral (10–25%) at maturity (Clark and Ogden, 1983). Subsequently, the peripheral vascular region (red-red zone) and the inner entirely avascular region (white-white zone) can be distinguished. The red-white zone is located between these two regions (Figure 1A). However, the healing capacity of each region is critically dependent upon the blood supply, thus leading to the avascular zone susceptible to post-traumatic and degenerative injuries (Arnoczky and Warren, 1982). For the treatment of meniscal tears, the current prevailing management trend is to maintain the integrity of meniscal tissue whenever possible (DeHaven, 1999; Turman and Diduch, 2008; Noyes and Barber-Westin, 2010). However, the meniscal lesions occurring within meniscal avascular zone are inevitably resected due to the limited intrinsic healing capacity (Kim et al., 2011; Jiang et al., 2017). Knee joint mechanical homeostasis is disrupted due to the interruption of meniscal tissue integrity after meniscal resection. The contact area between the femoral condyle and the corresponding tibial plateau is reduced (Zhang et al., 2019a). Therefore, cartilage contact stress is dramatically increased, leading to secondary cartilage deterioration or even osteoarthritis (OA) (Papalia et al., 2011). Hence, the research interest has been transferred toward facilitating meniscus avascular zone repair, where it is expected to maintain meniscal tissue integrity, prevent secondary cartilage degeneration and improve knee joint function.

To date, many clinical and basic studies have been performed to facilitate the healing of meniscus avascular zone injuries, such as the mechanical stimulation of the synovium and meniscus by rasping the parameniscal synovium and meniscal tear edges (Ritchie et al., 1998), the hoped introduction of blood supply by trephination between the red zone and white zone (Zhang et al., 1995), the addition of extrinsic fibrin clot (Henning et al., 1990) or the introduction of bone marrow cells and growth factors by bone marrow venting (Dean et al., 2017). While these studies are still in the early exploration stage, the mechanisms of action remain unclear. Moreover, there is a paucity of prospective randomized controlled trials and long-term follow-up studies to test clinical efficacy and safety, limiting their application in the clinic. Critically, the parameters affecting meniscal avascular zone repair are possibly multifactorial. The aim of this review is to clarify the possible mechanisms influencing meniscal avascular zone repair and the corresponding therapeutic strategies. In this review, we begin by summarizing the current surgical strategies for meniscal avascular zone injuries. We then discuss the possible impact of vascularization, inflammation, extracellular matrix (ECM) degradation and cellular component in meniscal avascular zone healing and the advances in therapeutic strategies. In our review, the current surgical therapies or new repair strategies related to vascularized zone were also included, as there were some reference values for the repair of avascular zone. Finally, perspectives for the future biological augmentations for meniscal avascular zone injuries will be given. Abbreviations used in this review are defined in Table 1.

**TABLE 1:** Abbreviations used.

Section	Abbreviations	Definition
Introduction	OA	Osteoarthritis
	ECM	Extracellular matrix
Replacement strategies	MAT	Meniscal allograft transplantation
	KOOS	Knee injury and Osteoarthritis Outcome Score
	SF-36	Short Form health survey-36 scores
	VAS	Visual analog score
	PCU	Polycarbonate urethane
	CMI	Collagen meniscus implant
	MRI	Magnetic resonance imaging
Promoting vascularization	VEGF	Vascular endothelial growth factor
	CTGF	Connective tissue growth factor
	CCN	Cellular communication network factor
	MFC	Meniscal fibrochondrocytes
	ESWT	Extracorporeal shockwave therapy
	TGF-β3	Transforming growth factor β3
	HGF	Hepatocyte growth factor
Inhibiting inflammation	SF	Synovial fluid
	RANTES	Regulated on activation, normal T cell expressed and secreted
	IL-6	Interleukin-6
	MCP-1	Monocyte chemotactic protein
	CCL	Chemokine ligand
	MMP	Mtrix metalloproteinase
	TIMP	Tissue inhibitor of metalloproteinase
	IL-1Ra	Interleukin 1 receptor antagonist
	bFGF	Basic fibroblast growth factor
	LAIRI	
	TMSB4X	A gene encoding a protein (TB4) that activates the expression of MMPS in fibroblasts (PubMed ID: 7114)
	IL 1B	Interleukin 1 beta
	IL1RN	Interleukin 1 receptor antagonist
	ILF2	interleukin enhancing binding factor 2
	NFKB1	Nuclear Factor of Kappa light polypeptide gene enhancer in B-cells 1
	IL10RB	Interleukin 10 receptor beta
	IL18BP	Interleukin 18 binding protein
	ILF3	Interleukin enhancing binding factor 3
	IL13RA	Interleukin 13 receptor, alpha 1
	BMP2K	Bone morphogenetic protein 2 inducible kinase
	IL10RB	Interleukin 10 receptor beta
	IFN-δ	Interferon δ
	MIP-1β	Macrophage inflammatory protein-1 beta
	TNF-alpha	Tumor necrosis factor alpha
	NSAIDs	Non-steroidal anti-inflammatory drugs
Inhibiting ECM degradation	ADAMTS	Aggrecanases
Cell-based therapy	ACL	Anterior cruciate ligament
	GAG	Glycosaminoglycans
	MSC	Mesenchymal stem cells

## Current Surgical Strategies

### Reduction Strategies

Reduction strategies in current use included tear closure with sutures or anchors and the trimming of torn segments (partial or total meniscectomy). Within the meniscal injuries, only a small percentage of meniscal injuries can be considered to be repairable regarding the tear pattern, tear position, vascularization, tear severity along with patient’s age (Figures 1B,C). For example, the vertical longitudinal tears within the “red-red” or “red-white” zone are often repairable with predictive good prognosis and patient satisfaction (Johnson et al., 1999; Fillingham et al., 2017). Tear closure was not a common treatment to meniscal tears in the avascular zone. Thus, the reduction strategy regarding tear closure in avascular zone was not introduced in this section. Even though the outcomes after meniscectomy still remained controversial in the field, such as a recent meta-analysis performed by Li (Li et al., 2020) concluded arthroscopic partial meniscectomy yielded better functional and pain outcomes compared to physical therapy in the short term until 12 months for degenerative meniscal tears, but, comparable results for pain and functional outcomes were observed at the 24-month follow-up time. Another meta-analysis of randomized controlled trials compared the outcomes of arthroscopic partial meniscectomy with conservative treatment in adults with non-obstructive meniscal tears. They concluded that small favorable results of physical function and pain were observed after arthroscopic partial meniscectomy within 6 months, but no differences were observed at 12 and 24 months (van de Graaf et al., 2016). However, meniscectomies either partial, subtotal or total are inevitably performed in the management of meniscal lesions that are mechanically unstable within “white-white” zone (Makris et al., 2011; Kwon et al., 2019). The partial meniscectomies are still the prevailing treatment options to remove the unstable and destroyed portion of the meniscal lesions within the avascular zone (Kim et al., 2011). Although the meniscectomies possess the advantages of rapid pain relief, faster return to sports or activities, accelerated rehabilitation protocols and lower revision surgery rate (Brelin and Rue, 2016; Feeley and Lau, 2018). The subsequent interruption of knee joint biomechanics caused by meniscectomies should not be neglected. A biomechanical research performed by Lee et al. (2006) clarified that the peak cartilage contact stresses increased dramatically and proportionally as the degree of meniscectomies. Paletta et al. (1997) demonstrated 50% reduction in cartilage contact area, resulting in 235–335% increase in peak contact stress after removal of total lateral meniscus in 10 cadaveric knees. Previous studies have demonstrated abnormal excessive mechanical load results in cartilage matrix degradation (Quinn et al., 2001; Thibault et al., 2002; Wilson et al., 2006) and chondrocyte apoptosis (D’Lima et al., 2001; Patwari et al., 2004). Thus, the secondary cartilage deterioration or even OA caused by abnormal biomechanics after meniscectomies should not be underestimated. The comparisons between advantages and disadvantages after meniscectomy were summarized in Table 2. Nevertheless, the meniscectomies are against the current prevailing management trend of maintaining the integrity of meniscal tissue whenever possible, a fact that supplies motivation for the development of novel interventions for meniscal avascular zone repair. What were stated in this section were as following: (i) meniscectomy was still the prevailing treatment to meniscal injuries in avascular zone; (ii) meniscectomy caused subsequent cartilage degeneration or even post-traumatic OA. (iii) New techniques for repairing meniscal lesions in the avascular zone were needed.

**TABLE 2:** Comparisons between advantages and disadvantages after meniscectomy.

Advantages	Disadvantages
Rapid pain relief	Destruction of meniscal tissue integrity
Faster return to sports or activities	Knee joint biomechanical imbalance
Accelerated rehabilitation protocols	Secondary cartilage deterioration or post-traumatic osteoarthritis
Lower revision surgery rate	

### Replacement Strategies

Meniscal substitution includes total and partial meniscal replacement based on the complaints and clinical symptoms of the patients. The options for total meniscal replacement include autologous (Goble et al., 1999; Johnson and Feagin, 2000) or allogenic (Kim et al., 2012; Marcacci et al., 2012; McCormick et al., 2014; Koh et al., 2018) transplants as well as the permanent artificial meniscal prosthesis (De Coninck et al., 2014; Vrancken et al., 2017). The available option for partial meniscal replacement includes scaffold-based meniscal substitute (Stone et al., 1997; Verdonk et al., 2011; Figure 1D).

#### Total Replacement

##### Autologous Substitutes

Several types of autologous tissue have been prepared as autograft for total meniscal replacement, such as tendon, fat or perichondral tissue owing to its superior safety and biocompatibility. In a previous study, the semitendinosus tendon autograft was used to reconstruct medial meniscus after total meniscectomies in a rabbit model. At 6 months post-surgery, the tendon grafts are incorporated with fibrochondrocytes, proteoglycan, type II collagen and radial type I collagen by histological evaluation. Moreover, no significant differences were observed between native and reconstructed menisci in terms of elastic modulus and hardness (Li et al., 2017). However, a longer follow-up animal study demonstrated the biomechanics of the tendon-meniscus were significantly worse compared with native menisci after total medial meniscal replacement with autologous patellar tendon for 12 months in a sheep model (Kohn et al., 1992). Similar worse biomechanical properties were revealed between reconstructed and native menisci when using infrapatellar fat pad or perichondral tissue (lower rib) autograft (Kohn et al., 1997; Bruns et al., 1998). Despite some promising results have been observed in animal study by using autograft as meniscal substitute, its application in clinic was limited due to the paucity of favorable clinical outcomes (Goble et al., 1999; Johnson and Feagin, 2000). This section stated that autologous substitutes did not demonstrate sufficient beneficial effects on cartilage protection and regenerating meniscal tissues in the avascular zone.

##### Allogenic Substitutes

The allogenic substitute for total meniscal replacement refers to the Meniscal allograft transplantation (MAT). The MAT still remains to be the standard-of-care option for those patients with symptomatic post-meniscectomy syndrome after subtotal or total meniscectomies. The MAT is indicated for patients with stable knee, aligned lower limb and, at most, early knee OA (Verdonk et al., 2013). The detailed indications and contraindications for MAT are summarized in Table 3. Overall, the patient reported outcomes including symptom relief and function improvement after MAT improved significantly regardless of medial or lateral MAT and fixation techniques (Kim et al., 2012; Marcacci et al., 2012; Rosso et al., 2015; Koh et al., 2018). A long-term follow-up study (mean follow up time: 152 months; range, 112–216 months) evaluated thirty patients who underwent MAT from the perspectives of Knee injury and Osteoarthritis Outcome Score (KOOS), Lysholm, Tegner, Short Form-36 scores (SF-36) as well as visual analog score (VAS) for pain. The results demonstrated MAT resulted in significant improvements in patient satisfaction, pain relief and functional scores at long term follow-up in spite of an increase in joint space narrowing (Vundelinckx et al., 2014). However, the intrinsic limitations that may affect clinical outcomes or applications should not be neglected. Firstly, the size of meniscal allograft has to fit properly with the recipients. Oversizing or undersizing of more than 10% of the original meniscal size both affect the desired benefits, accounting for the failure of MAT or subsequent joint degenerative changes (Dienst et al., 2007). Secondly, the availability of meniscal allograft could not be achieved in many countries, thus restricting its broad application in clinic. This section stated that some limitations were still emerging for MAT, such as disease transmission, size unmatching, acquisition and cartilage protection, despite its advantages in symptom relief and function improvement.

**TABLE 3:** Indications and contraindications for scaffold-based meniscal substitutes and meniscal allograft transplantation.

	Scaffold-based meniscal substitution	Meniscal allograft transplantation
Indications	Clinical symptoms	Clinical symptoms
	s/p extensive partial meniscal resection	s/p sub-/total meniscal resection
	Stable meniscal rim	Insufficient meniscal rim
	Intact meniscal roots	Insufficient meniscal roots
	Chronic partial meniscal defect	
Contraindications	Age: > 50 years^a^	Age: > 50 years^a^
	BMI: > 35^a^	BMI: > 35^a^
	Insufficient meniscal rim	Outerbridge grade III, IV
	Insufficient meniscal roots	Fairbank grade > 2
	Knee instability	Joint space narrowing
	Limb malalignment	Knee instability
	Allergies to animal derived products	Limb malalignment
	ICRS grade > 3	Active infection
	Active infection	Autoimmune diseases
	Autoimmune diseases	Inflammatory arthritis
	Inflammatory arthritis	Smoker^a^
	Smoker^a^	

*ICRS, International cartilage repair society; s/p, status post.*

*^a^Relative contraindication.*

##### Artificial Substitutes

The currently available permanent artificial total meniscal prostheses under investigation, clinical trials or clinical use are mainly composed of polycarbonate-urethane (PCU). Anatomically and non-anatomically shaped meniscal prostheses are included according to the morphology (Zur et al., 2011; De Coninck et al., 2014; Vrancken et al., 2016, 2017; Shemesh et al., 2020). A few animal studies have confirmed the chondroprotective effect of artificial meniscal prostheses. A non-degradable, anatomically shaped artificial implant, composed of Kevlar-reinforced PCU was shown to delay or prevent osteoarthritic changes after implantation of 6 months post-surgery in a sheep model (Zur et al., 2011). Vrancken et al. (2016) demonstrated the biomechanical performance of a novel anatomically shaped PCU total meniscal implant was similar to meniscal allograft. Moreover, the same PCU total meniscal implant functioned similarly to MAT in chondroprotective potential, where it was expected the novel PCU implant may have the potential to be an alternative for meniscal allograft (Vrancken et al., 2017). NUsurface^®^ Meniscus Implant (Active Implants LLC, Memphis, TN, United States), a polyethylene reinforced PCU prosthesis with the characteristics of non-anatomically discoid-shaped, free floating and non-anchored was designed for total meniscal replacement (De Coninck et al., 2014; Shemesh et al., 2020). A biomechanical study performed in cadaveric knees demonstrated the NUsurface^®^ Meniscal Implant restored the average and peak contact pressure to 93 and 92% of its pre-meniscectomy status, respectively (Shemesh et al., 2020). A pilot study including three patients was performed to assess the knee kinematics after implantation of NUsurface^®^ Meniscus Implant. No differences were observed between the implanted knees and contralateral healthy knees in terms of knee and meniscal kinematics except for anterior-posterior movement of meniscus (De Coninck et al., 2014). There is a paucity of rigorous evidence based clinical data supporting its clinical effectiveness and safety, despite the permission for clinical use has been approved in Europe and Israel in 2008 and 2011, respectively. This section stated that the cartilage protection potential of artificial meniscal implant still needed rigorous evidence.

#### Partial Replacement

The currently commercial scaffold-based partial meniscal substitutes include the collagen meniscus implant (CMI; Stryker Corporation, Kalamazoo, MI, United States) (Stone et al., 1997), composed of type I collagen derived from bovine Achilles tendons and polyurethane polymeric implants (Actifit, Orteq Sports Medicine Ltd., London, United Kingdom) (Verdonk et al., 2011), composed of polycaprolactone (80%) and polyurethane (20%). The scaffold-based partial meniscal substitutes are indicated for the patients with extensive partial meniscal resection, stable meniscal rim, intact meniscal roots and limited cartilage damage (van Tienen et al., 2009). The detailed indications and contraindications for scaffold-based meniscal substitution are listed in Table 3. A prospective randomized, controlled, multicenter, comparative clinical trial demonstrated the patients receiving CMI regained significantly more activity than did the patients treated with partial meniscectomy (Rodkey et al., 2008). A minimum of 10 years’ follow-up study showed significant pain relief and functional improvement after CMI implantation. The safety and lower rate of failure after CMI implantation have been confirmed despite the reduction in implant size was present (Monllau et al., 2011). Similarly, the Actifit implant demonstrated favorable prognosis regarding patient reported outcomes (KOOS, KSS, UCLA Activity Scale, VAS for pain) and MRI manifestations (Schüttler et al., 2016). Even though the scaffold-based partial meniscal substitutes showed some promising clinical outcomes, several drawbacks were still present. Firstly, the regenerated new meniscus-like tissue could not represent native meniscus; secondly, the capacity to prevent OA progression still needs to be proved; thirdly, the proper placement of implant is difficult to be achieved by arthroscopy (Kwon et al., 2019). This section stated that some limitations were still emerging for partial meniscal replacement, such as the capacity to reconstruct meniscal tissues, cartilage protection and precise placement under arthroscope. Hence, there is a great need to develop novel therapies to facilitate meniscal repair.

## New Strategies

### Promoting Vascularization

From clinical practice, the richly vascularized “Red-Red” zone and relatively vascularized “Red-White” zone have superior healing capacities compared with the avascular “White-White” zone. The lack of sufficient vascularity in the avascular zone is assumed to be the key factor in the absence of healing in this zone (Arnoczky and Warren, 1983). In this section, the strategies that are expected to improve angiogenesis and the healing results of meniscal injuries in the avascular zone are reviewed. Previous studies investigating the potential effects on avascular meniscal tissue repair by promoting vascularization are summarized in Table 4.

**TABLE 4:** Summary of studies regarding promoting vascularization in the healing of avascular meniscal lesions.

Model	Duration	Treatment	Outcome	Reference
**Meniscal tear without intervention**				

Rabbit	1–10 weeks	Vascular meniscal tear (*n* = 20) Avascular meniscal tear (*n* = 20) No surgery (*n* = 5)	Progressive vascularization and complete healing; increased endogenous VEGF No vascularization or healing; highest endogenous VEGF NA	Becker et al., 2004
Rabbit	1–20 weeks	Avascular meniscal tear (*n* = 36) No surgery (*n* = 6)	No evidence of healing, VEGF expression peaked at 14 days NA	Ruiz Ibán et al., 2014

**Meniscal tear with VEGF supplementation**				

Sheep	6 weeks	VEGF + coated suture (*n* = 6) coated suture (*n* = 6)	6/6 no healing 3/6 partial healing; 3/6 complete healing	Petersen et al., 2005
Sheep	8 weeks	VEGF + coated suture (*n* = 6) Coated suture (*n* = 6) Uncoated suture (*n* = 6)	1/6 partial healing; 5/6 no healing 1/6 complete healing; 1/6 partial healing; 4/6 no healing 3/6 partial healing; 3/6 no healing	Kopf et al., 2010
Sheep	8 weeks	Suture + trephination (*n* = 4) Matrigel^TM^ (*n* = 4) VEGF (*n* = 4) Cultured chondrocytes (*n* = 4) Hyaluronic acid (*n* = 4) BMP-7 (*n* = 4)	No healing No healing No healing Fibrous tissue healing No healing Fibrous tissue healing	Forriol et al., 2015

**Meniscal tear with CTGF supplementation**				

Rabbit	10 weeks	CTGF + fibrin glue	complete healing with capillaries	He et al., 2011

**Meniscal tear with other bioactive factors supplementation**				

Meniscal explant	4 weeks	HGF + PDGF	Meniscal defect was repaired with organized collagen	Bhargava et al., 2005
Meniscal explant	4 weeks	endothelin-1	superior integrative healing of meniscal implants	Yuan et al., 2015

*BMP-7, bone morphogenic protein-7; NA, not applicable.*

#### Vascular Endothelial Growth Factor

The angiogenic growth factor, vascular endothelial growth factor (VEGF), is an endothelial cell mitogen with the potential to enhance angiogenesis and microvasculature permeability (Ferrara, 2004). VEGF has been demonstrated to play an important role in vascular growth, such as the musculoskeletal system (Pufe et al., 2001), as well as the vascular proliferation in physiologic and pathologic conditions. To date, the expression of at least 12 pro- and anti-angiogenic isoforms of VEGF have been documented (Nowak et al., 2008).

Two observational studies demonstrated an increase of VEGF following the preparation of meniscal injuries in rabbits (Becker et al., 2004; Ruiz Ibán et al., 2014). One observational study demonstrated and increase of VEGF-A at the level of mRNA expression in the meniscal avascular zone on the 14th day after the preparation of longitudinal lesion in the avascular zone of the anterior horn of medial meniscus. The expression level of VEGF-A returned to the base at 120 days (Ruiz Ibán et al., 2014). Another observational study demonstrated that the expression of VEGF reached the highest at the level of VEGF protein and VEGF mRNA in the avascular area after meniscal lacerations in the first week (Becker et al., 2004). Although increased expression of intrinsic VEGF after meniscal injuries has been demonstrated, meniscal healing in the avascular zone fails, indicating that intrinsic VEGF is unlikely to stimulate healing. Thus, other studies attempted to promote meniscal healing in the avascular zone by local administration of exogenous VEGF. However, no healing was observed in these studies (Petersen et al., 2005; Kopf et al., 2010; Forriol et al., 2015). The following three possible reasons may account for the failure of healing. First, the administration of VEGF did not achieve the therapeutic dose to promote healing. Second, tissue specificity or specific combinations of VEGF isoforms may be needed to achieve vascularization and healing due to the existence of multiple VEGF isoforms. Third, the presence of intrinsic antagonists within the avascular zone has the potential to inhibit vascularization. Therefore, future investigations should be performed to identify potential antagonists in the avascular zone as well as the optimal type and dose of VEGF isoform. This will allow researchers to better evaluate the potential use of VEGF for enhancing vascularization and healing meniscal lesions in the avascular zone.

#### Connective Tissue Growth Factor

Connective tissue growth factor (CTGF), a member of the CCN protein family, functions as a signaling protein for the coordination of the synthesis of ECM (Rachfal and Brigstock, 2005). CCN proteins have been reported to be major regulators of chondrogenesis, angiogenesis, and fibrogenesis. CTGF has been demonstrated to enhance the proliferation of vascular endothelial cells *in vitro* and angiogenesis *in vivo* (Shimo et al., 1999). A previous study demonstrated that supplementation of rabbit meniscal fibrochondrocytes (MFCs) with CTGF *in vitro* resulted in increased expression of type 1 and 2 collagen and VEGF mRNA. The avascular meniscal defect was completely filled with type 1 and 2 collagen as well as newly formed capillaries (He et al., 2011). Another *in vitro* study reported enhanced alignment of collagen fibers, fibrocartilaginous matrix elaboration and mechanical properties after supplementation with high-dose CTGF (1,000 ng/ml) and a slowed release rate of TGFβ3 (0.29 ± 0.1 ng/day) in a bovine meniscal explant tear model (Tarafder et al., 2019). Recently, Hashimoto et al. (2019) demonstrated that extracorporeal shockwave therapy (ESWT) could promote the healing of meniscal lesions in the avascular zone of rats. The mRNA level of CTGF at the tear site was upregulated significantly after ESWT treatment, further demonstrating the potential role of CTGF in avascular meniscal healing. A previous study finished by Nakagawa et al. (2019) supported the application of CTGF in meniscal healing. Nakagawa et al. (2019) demonstrated 3-dimensional (3D) printed anatomical scaffolds loaded with CTGF and transforming growth factor β3 (TGF-β3) achieved meniscal regeneration in an ovine model at 12 months follow-up. Even though vascularization was not overtly demonstrated, CTGF likely enhanced VEGF levels within the meniscus and contributed to the healing of avascular meniscal lesions.

#### Other Bioactive Factors

Other bioactive factors, such as hepatocyte growth factor (HGF) and endothelin-1, have also been used with the expectation of enhancing avascular meniscal healing. A previous study assessed the potential role of HGF in enhancing angiogenesis in avascular engineered meniscal tissue when implanted into the subcutaneous pouch of immunosuppressed mice (Hidaka et al., 2002). More blood vessels could be observed in meniscal implants overexpressing HGF, suggesting the potential role of HGF in promoting vascularization in avascular meniscal lesions. Another two *in vitro* studies also demonstrated the potential role of HGF in promoting meniscal cell proliferation and migration to stimulate meniscal healing (Bhargava et al., 1999, 2005). Endothelin-1 produced by endothelial cells is a potent vasoactive molecule (Agapitov and Haynes, 2002). Endothelin-1 has also been reported to stimulate angiogenesis in various tissues, including cartilage (Kinoshita et al., 1995; Carmeliet, 2000). Only one *in vitro* study demonstrated that the increased migration of bovine MFCs was dose-dependent when co-cultured with endothelial cells. Moreover, endothelin-1 was identified to stimulate meniscal cell migration as well as the superior integrative healing of meniscal implants (Yuan et al., 2015). However, the investigation of HGF and endothelin-1 in facilitating meniscal angiogenesis and healing is still in its initial stage. Further *in vitro* and *in vivo* studies are needed to assess their potential role in this field.

This section stated that small favorable results supported that targeting vascularization could facilitate vascular proliferation and meniscal repair in the avascular zone. We think that several rigorous questions should not be neglected. Firstly, Chondromodulin-1 and endostatin in meniscal avascular zone have been validated to suppress vascularization (O’Reilly et al., 1997; Pufe et al., 2004; Miura et al., 2010; Fujii et al., 2013). Thus, it remained a question whether the function of VEGF, CTGF or other aforementioned pro-angiogenic factors to promote vascularization could work. Secondly, the degenerated or aged menisci and hyaline cartilage were often accompanied by increased invading blood vessels and decreased Chondromodulin-1 expression (Kitahara et al., 2003; Fransès et al., 2010; Ashraf et al., 2011). However, Chondromodulin-1 is critical in promoting chondrocyte proliferation and maintaining chondrocyte phenotype (Hiraki and Shukunami, 2000). The MFC in meniscal avascular zone demonstrated hyaline chondrocyte-like phenotype. It was speculated that the meniscal tissue degenerated, once vascularization succeeded. Thus, it necessitated further assessment that facilitating avascular meniscal tissue repair by promoting vascularization.

### Inhibiting Inflammation

After tissue injury, a series of inflammatory reactions can be triggered, and the long-term inflammatory response not only interferes with the repair process of the damaged tissue but also triggers a series of cascade reactions. In this section, the inflammatory reaction and its effect on meniscal healing after meniscal lesions as well as the potential effect on promoting meniscal repair after antagonizing inflammatory factors will be reviewed.

#### Inflammatory Reaction After Meniscal Lesions

In a previous study focused on alterations in synovial fluid (SF) biomarker levels, the concentrations of 10 biomarkers (RANTES, IL-6, MCP-1, CCL4, MMP-3, TIMP-1, TIMP-2, IL-1Ra, VEGF, and bFGF) were measured in synovial fluid collected from both the meniscal injured knee and the contralateral uninjured knee at the time of surgery using a multiplex magnetic bead immunoassay (Clair et al., 2019). The concentrations of four pro-inflammatory biomarkers (IL-6, MCP-1, CCL4, and MMP-3) in meniscal injured knees increased significantly compared to those of the contralateral asymptomatic knees. Another previous study characterized the gene expression profiles in SF concentrating on inflammation and arthritis related genes at different time points after meniscal injuries (Vance, 2014). The included patients were categorized into short injury duration (≤2 months) and long term (≥3 months) injury duration cohorts. The gene expression profiles in cell pellets and supernatant were characterized by using microarray and RNA-Seq. The results showed that LAIRI (an immune inhibitory receptor), IL-10, and TMSB4X (a potential activator of MMP expression) were highly expressed significantly in both populations. Regarding the differences between cohorts, 10 genes (IL 1B, IL1RN, ILF2, NFKB1, IL10RB, IL18BP, ILF3, IL13RA, BMP2K and IL10RB) primarily correlated to the inflammation were upregulated significantly in the long injury duration cohort compared to the short injury duration cohort. Furthermore, the inflammatory cytokines in the SF, such as IFN-δ, IL-6, MCP-1, MIP-1β, were involved in pain with symptomatic meniscal lesions (Cuellar et al., 2009). Also, the cytokines of MCP-1, IL-6 and IL-8, were still existing in SF several months after meniscal injuries (Larsson et al., 2015; Bigoni et al., 2017). These studies demonstrated that the process involved in inflammation related to meniscal lesions progressed over time. However, the reported upregulation of proinflammatory cytokines after meniscal injuries has mainly focused on SF. The exact expression profiles in injured meniscal tissues need further investigation. This section stated that pro-inflammation existed within the knee joint, demonstrating upregulated expression of pro-inflammatory factors.

#### Effect of Inflammation and Anti-Inflammation on Meniscal Healing

A previous study assessed the influence of proinflammatory cytokines, including interleukin-1 (IL-1) or tumor necrosis factor alpha (TNF-alpha), on meniscal repair using meniscal explants harvested from the peripheral vascularized porcine meniscus (Hennerbichler et al., 2007). Weak cell accumulation and mechanical strength were observed in the presence of IL-1 or TNF-alpha, while superior healing outcomes were observed in the absence of pro-inflammatory cytokines, demonstrating the spontaneous healing response could be hindered by pro-inflammatory cytokines. Another previous *in vitro* study also clarified the inhibitory effect of IL-1 on the integrative repair of the meniscus when supplemented with IL-1alpha acutely (100 pg/mL for 1 or 3 days) or chronically (100 pg/mL for entire culture duration). Inferior shear strength, cell viability and tissue integration were present after exposure to IL-1 (Wilusz et al., 2008). The negative effect of pro-inflammatory cytokines on the healing process of meniscal lesions was also demonstrated in other studies (McNulty and Guilak, 2008; Riera et al., 2011; McNulty et al., 2013). However, the inhibitory effects of pro-inflammatory cytokines could be reversed by the relative antagonists. McNulty et al. (2007) demonstrated that the supplementation of antagonists for IL-1 or TNF alpha enhanced the integrative repair of meniscal explant in an inflammatory microenvironment by using an *in vitro* meniscal model system. Although the aforementioned studies mainly focused on the vascularized meniscal tissues, the negative effects of inflammation or reversed inhibitory effect after antagonist supplementation could not be excluded for avascular meniscal tissue. Future *in vitro* and *in vivo* studies should be performed to evaluate the potential effect of inflammation and anti-inflammation on the repair of avascular meniscal lesions. Moreover, the potential benefit of anti-inflammation, such as non-steroidal anti-inflammatory drugs (NSAIDs) or antagonists for pro-inflammatory cytokines, on avascular meniscal tissue repair is worth investigating. In this section, we stated that inflammation affected meniscal repair negatively and anti-inflammation may be a candidate to facilitate meniscal repair in the avascular zone.

### Inhibiting Extracellular Matrix Degradation

Under physiological conditions, a dynamic balance was noted between ECM synthesis and degradation (Goldring and Otero, 2011). However, loss of collagen and proteoglycan from meniscal ECM was presented in the presence of meniscal degeneration or injuries (Herwig et al., 1984). During ECM degradation, metalloproteinases (MMPs) and aggrecanases (ADAMTS) are the primary two types of degradative enzymes responsible for the degradation of meniscal ECM components (Goldring and Otero, 2011). Thus, in this section, ECM degradation and its effect on meniscal healing after meniscal lesions as well as the potential effect on promoting meniscal healing after inhibiting degradative enzymes will be reviewed.

**FIGURE 2:**
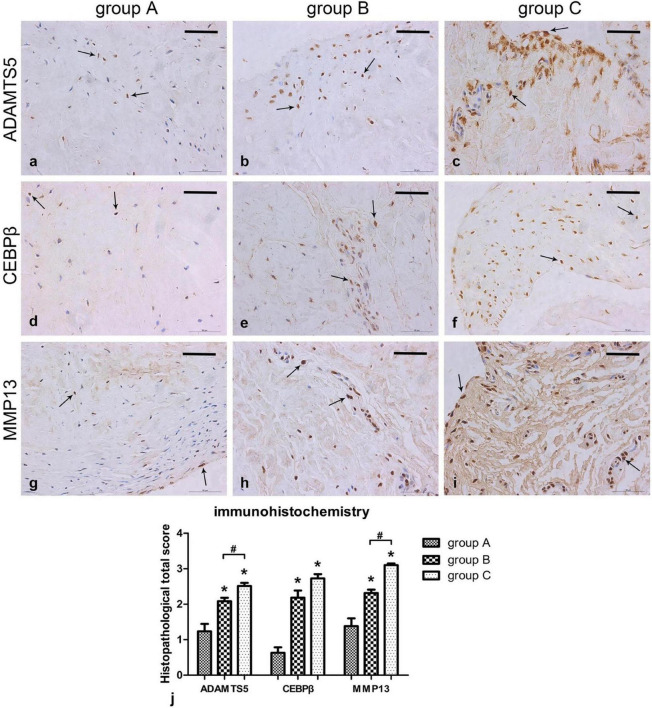
Immunohistochemical results showing ADAMTS5, CEBPβ, and MMP13 levels in the meniscus. **(a–i)** Sections were counterstained with haematoxylin, representative immunohistochemical positive cells are marked with arrows, Scalebar: 50 μm. **(j)** Immunohistochemistry graph showing total histopathological score in group A (normal meniscus), group B (simple meniscal tear), and group C (meniscal tear with concomitant ACL tear), taken as the product of the specific reactivity (IR) and proportion of positive cells. The values are given as the mean and the standard error of mean. Within a given group, significant difference (*p* < 0.05) compared with group A is denoted with (*). Significant differences (*p* < 0.05) between group B and group C are marked with (#). Representative results from at least three samples in each group are shown. This figure was adapted permission from Long et al. (2019).

A previous study demonstrated that the concentration of MMP-3 in the SF of symptomatic knees with meniscal injuries was higher than that of the contralateral asymptomatic knees (Clair et al., 2019). MMP-3 is a well-known stromelysin that degrades collagen, proteoglycans, fibronectin, laminin, and elastin and responsible for activating other MMPs (Emonard and Grimaud, 1990; Mehraban et al., 1994). TIMP is a family of protease inhibitors regulating the catabolic activity of MMPs (Ries, 2014). Moreover, the ratio of MMP-3 to TIMP-1 was also elevated after meniscal lesions, demonstrating imbalance between proteases and protease inhibitors after meniscal injury. Liu et al. (2017) also demonstrated the total MMP activity in SF was increased 25-fold in meniscal injury populations compare to the controls. The upregulated expression of MMPs or ADAMTS in SF after meniscal lesions were demonstrated in many other studies (Brophy et al., 2012; Carter et al., 2015; Roller et al., 2016). Another previous study directly demonstrated the elevated expression of MMP13 and ADAMTS5 in injured meniscal tissues using real-time polymerase chain reaction along with immunohistochemistry (Long et al., 2019; Figure 2). McNulty et al. (2009) assessed the potential promoting effect of broad-spectrum MMP inhibitor GM 6001 on meniscal healing in the presence of IL-1 using an *in vitro* meniscal repair model system. When treated with MMP inhibitor, they observed a decrease in total MMP activity in the culture media, an increase in the shear strength of repair, and enhancement in tissue repair in the injury interface even though in the presence of IL-1, suggesting IL-1 suppressed meniscal healing through upregulating MMP activities. Further *in vivo* studies should be performed to evaluate the potential effect of directly inhibiting ECM degradative enzymes or proinflammatory cytokines, such as IL, or in combination on repairing avascular meniscal injuries. In this section, we stated that inhibiting ECM degradative enzymes or pro-inflammatory cytokines may be a candidate for repairing avascular meniscal tissue lesions.

### Cell-Based Therapy

The cellular component plays a critical role in tissue repair and regeneration. In tissue engineering, such as meniscal tissue engineering or cartilage tissue engineering, intrinsic or extrinsic cellular components are indispensable (Makris et al., 2011; Kwon et al., 2019). In this section, cell viability and recruitment after meniscal injuries as well as current cell-based therapies in meniscal repair are reviewed.

#### Inhibiting Apoptosis

Uysal et al. (2008) demonstrated the existence of apoptosis in the traumatic and degenerative tears of human meniscus with normal meniscal tissues harvested from cadavers and traumatic or degenerative meniscal tissues harvested from patients demanding arthroscopic operations both below the age of 40. More apoptotic cells were investigated in the traumatic or degenerative meniscal tissues than the normal meniscal tissues. Another previous study assessed the acute meniscal cell viability after closed-joint knee injury in a lapine model. A significant acute decrease in meniscal cell viability was demonstrated 24 h post-injury following meniscus tears caused by traumatic impaction (Killian et al., 2014). Hashimoto et al. (1999) evaluated the degree of apoptosis in degenerated menisci following anterior cruciate ligament (ACL) transection. Apoptosis was prominent in the more degenerated regions of menisci. The proliferating meniscal cell clusters emerged when subjected to ACL transection, however, most of the cells in the clusters showed apoptosis. The subsequent destructive processes secondary to cell death may be presented in meniscal tissues. Firstly, the decrease in cellularity impaired the intrinsic potential of maintaining and remodeling meniscal tissue. Secondly, cell apoptosis or necrosis could contribute to the degradation of ECM. For instance, the intracellular enzymes, such as lysosomal glycosidases or metalloproteinases released from the necrotic cells could lead to ECM degradation. On the other hand, apoptotic body originating from apoptotic cell, a membrane-enclosed unit containing cellular components could result in matrix degradation. Thus, alleviating cell death may be the potential target for repairing meniscal lesions. Another two previous studies from the same research team demonstrated the beneficial effect of Poloxamer 188 (P188), a non-ionic surfactant showing promise in preventing cell death by maintaining the integrity of cell membranes, on preserving meniscal cell viability, GAG content and mechanics after meniscal lesions caused by abnormal impaction (Coatney et al., 2015; Narez et al., 2021). These two previous studies showed the potential benefit of inhibiting cell apoptosis after meniscal injuries on repairing meniscus. Thus, in the future, it is worth investigating the potential effect of inhibiting meniscal cell apoptosis after meniscal injuries on repairing meniscal avascular zone tears, such as local administration of apoptosis inhibitors.

#### Enhancing Cell Recruitment

Cell recruitment is a critical process in tissue repair. However, a previous study has clarified that the dense organized ECM in mature menisci inhibited the migration of adult meniscal cells due to the imposed biophysical barriers by mature ECM, which was opposite in immature menisci ECM or partially enzymatically digested mature menisci (Qu et al., 2018). Thus, it is difficult to achieve meniscal healing through the migration of intrinsic mature meniscal cells, especially in the presence of cell apoptosis in injured meniscal tissues. Qu et al. (2013) developed a biomaterial-mediated delivery of degradative enzymes to improve meniscal integration and repair. They fabricated the electrospun poly (ethylene oxide) (PEO) nanofibers to carry collagenase and then implanted into the tear interface using an adult bovine meniscal explant model. They concluded that the released collagenase degraded the compact meniscal ECM and expedited meniscal cell migration to the wound edge and meniscal tissue remodeling. They further evaluated the repair potential of this biomaterial-mediated delivery system of degradative enzymes on full-thickness longitudinal meniscal tear in the avascular zone using an ovine model. They found clear apposition of the margins and tissue formation both within and surrounding the scaffold across the length and height of the tear indicating robust healing response (Qu et al., 2015). These studies stated meniscal repair in the avascular zone could be achieved by recruiting remaining meniscal cells through degrading dense meniscal ECM by locally administrating collagenase, which provided an innovative approach to facilitate avascular meniscal tissue repair.

#### Mesenchymal Stem Cells-Based Therapy

Mesenchymal stem cells (MSCs) have demonstrated superiority in tissue repair and regeneration, especially in constructing tissue-engineered menisci (Kremer et al., 2017; Zhang et al., 2017, 2019b). Therefore, most studies stimulated meniscal repair through applying extrinsic MSC. Nakagawa et al. (2015) demonstrated the beneficial effect of transplantation of synovial MSC on promoting repair of the meniscal longitudinal tears in the avascular zone of meniscus in pigs. Another study clarified the beneficial effect of transplantation of allogenic synovial MSC on promoting meniscal healing using a novel pig meniscus injury model by punctuated 200 times using a 23G needle (Ozeki et al., 2021). Another previous study evaluated the potential effect of undifferentiated autologous bone marrow mesenchymal stem cells (BMMSCs) seeded onto a collagen scaffold (MSC/collagen-scaffold) on repairing torn avascular meniscal tissues in a single center, prospective, open-label first-in-human safety study (Whitehouse et al., 2017). Among the included five patients with avascular meniscal tears, three were reported to be asymptomatic after treatment until 24 months with no signs of recurrent tears form MRI as well as significant improvement in knee function. Another two patients required meniscectomies due to retear or non-healing at approximately 15 months after treatment. However, a multi-center, prospective, randomized controlled trails (RCT) is needed to further testify its efficacy on promoting avascular meniscal repair. Intrinsic MSCs, such as synovial MSC, adipose-derived MSC and synovial fluid-derived MSC within the knee joint are abundant and potent in repairing injured tissues in the knee joint. One previous study demonstrated locally administered magnesium could promote meniscal repair through recruiting synovial fluid-derived MSC (Zhang et al., 2019c). Further studies should be performed to compare the potency of intrinsic and extrinsic MSCs as well as the type of MSC in promoting avascular meniscal healing.

In this section, we stated that saving the intrinsic meniscal cells by inhibiting cell apoptosis after meniscal injuries or facilitating recruitment of remaining meniscal cells and extrinsic cells including motion of other tissues derived reparative cells or injection of reparative cells may facilitate avascular meniscal tissue repair.

## Conclusion

**FIGURE 3:**
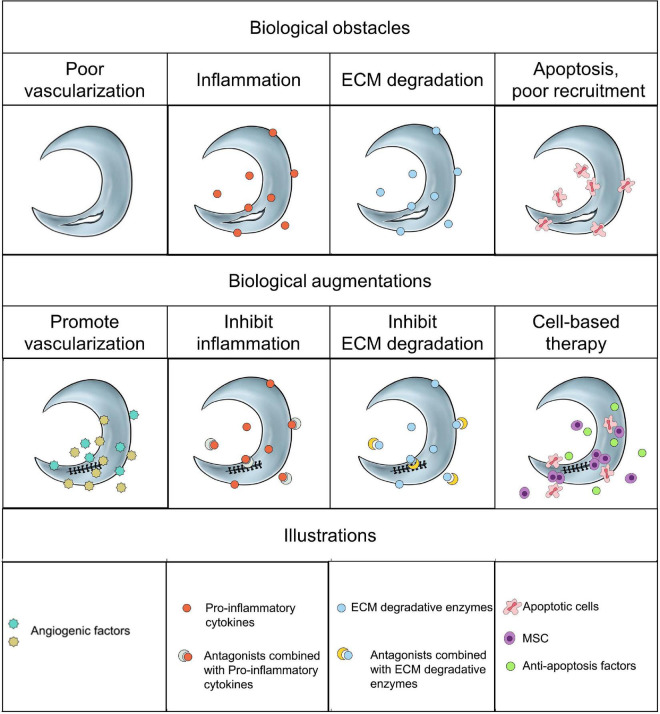
The schematics of the current biological obstacles and biological augmentations for meniscal avascular zone repair.

Current surgical strategies for meniscal avascular zone lesions remained to be insufficient to prevent the development of OA, thus accelerating the development of alternative biological augmentations to promote meniscal healing. The biological mechanisms affecting meniscal avascular zone healing demonstrated multiple, such as poor vascularization, inflammation, ECM degradation, cell apoptosis and poor cell recruitment. For vascularization, the combination of angiogenic growth factors (e.g., VEGF, CTGF, HGF) may be more effective in promoting vascularization. However, the degenerated or aged menisci were often accompanied by increased vascularization. Thus, it necessitated further assessment that facilitating avascular meniscal tissue repair by promoting vascularization. The presence of inflammation and ECM degradation certainly demonstrated negative effect on meniscal healing. Inhibiting pro-inflammatory cytokines and ECM degradative enzymes to build a favorable environment is critical for meniscal healing process. More apoptosis and decreased cell viability were demonstrated in the traumatic or degenerative meniscal tissues than normal meniscal tissues at the early phase after injuries. Alleviating cell apoptosis and increasing intrinsic meniscal cell viability may be positive in promoting meniscal avascular zone repair. Moreover, the migration of adult meniscal cells was inhibited by the dense organized ECM in mature menisci due to the imposed biophysical barriers by mature ECM. Preparing porous tunnels at the tear interface by locally administrating collagenase and subsequent enhanced meniscal cell migration provided an innovative approach to facilitate avascular meniscal tissue repair. Enhancing the intrinsic healing potential of avascular meniscal tissue by recruiting other tissues derived reparative cells within the knee joint, such as synovium derived MSC, adipose derived MSC, provided a novel approach. Also, the transplantation of extrinsic MSC has shown promise in stimulating avascular meniscal tissue repair, but a multi-center, prospective, randomized controlled trails (RCT) is needed to further testify its efficacy. The schematics of the current biological obstacles and biological augmentations for meniscal avascular zone repair were shown in Figure 3.

However, additional factors i.e., gender, age, weight, activity requirement, pre-existing conditions and concomitant injuries also play important roles in meniscal repair. Individual-based treatment approach represents the future direction. Moreover, the rehabilitation approach is also critical for the repair of meniscus especially when the new tissues are deposited to bridge the tear at the early phase. Thus, besides biological augmentations, the aforementioned additional factors should also be considered.

## Author Contributions

WY, WD, JC, XH, and YA contributed to conceptualization, methodology, validation, formal analysis, investigation, data curation, and original draft and revise. WY, WD, JC, YF, TW, FZ, and JZ contributed to methodology, validation, data curation, and original draft and revise. XH and YA contributed to conceptualization, original draft and revise, review and editing, and supervision.

## Conflict of Interest

The authors declare that the research was conducted in the absence of any commercial or financial relationships that could be construed as a potential conflict of interest.

## Publisher’s Note

All claims expressed in this article are solely those of the authors and do not necessarily represent those of their affiliated organizations, or those of the publisher, the editors and the reviewers. Any product that may be evaluated in this article, or claim that may be made by its manufacturer, is not guaranteed or endorsed by the publisher.
